# Validation of the Reliability of Machine Translation for a Medical Article From Japanese to English Using DeepL Translator

**DOI:** 10.7759/cureus.17778

**Published:** 2021-09-06

**Authors:** Yosuke Takakusagi, Takahiro Oike, Katsuyuki Shirai, Hiro Sato, Kio Kano, Satoshi Shima, Keisuke Tsuchida, Nobutaka Mizoguchi, Itsuko Serizawa, Daisaku Yoshida, Tadashi Kamada, Hiroyuki Katoh

**Affiliations:** 1 Department of Radiation Oncology, Kanagawa Cancer Center, Yokohama, JPN; 2 Department of Radiation Oncology, Gunma University Graduate School of Medicine, Maebashi, JPN; 3 Department of Radiology, Saitama Medical Center, Jichi Medical University, Omiya, JPN

**Keywords:** reliability, machine translation, back translation, deepl translator, japanese to english

## Abstract

Background

The reliability of DeepL Translator (DeepL GmbH, Cologne, Germany) for the translation for medical articles has not been verified yet. In this study, we investigated the accuracy of machine translation from Japanese to English for a medical article using the DeepL Translator.

Methodology

The subject of this study was an English-language medical article translated from Japanese, which had already been published. The original Japanese manuscript was translated into English using DeepL Translator. The translated English article was then back-translated into Japanese by three researchers. In turn, three other researchers compared the back-translated Japanese sentences with the original Japanese manuscript and calculated the percentage of sentences that retained the intended meaning.

Results

The mean ± standard deviation of the match rate for the entire article was 94.0 ± 2.9%. The match rate in the Results section was significantly higher than that in the other sections; while the match rate in the Materials and Methods section was significantly lower than the rate in the other sections. Compound sentences and sentences with an unclear subject and predicate appeared to be significant predictors for mismatched translation.

Conclusions

The translation for a medical article from Japanese to English was performed accurately by DeepL Translator.

## Introduction

Approximately 50% of all international medical articles originate from non-English-speaking countries [1]. The share of medical articles published from Japan is only 6% and is gradually declining [1]. Writing medical articles in English is one of the obstacles faced by non-English-speaking researchers.

Machine translation (MT) is a system that automatically translates one language into another. The quality of MT has been improving, and it is widely used in various fields, such as business and translation services [2]. MT reduces the time required for translation by 95% of that required for manual translation [3]. Conventional MT uses the statistical machine translation (SMT) framework [4]. This approach employs a large amount of parallel text of the target language pair to train SMT models. Recently, MT systems employing different approaches, such as neural networks, have been developed [2]. Several studies have demonstrated that MT using neural networks shows higher translation accuracy in comparison with conventional SMT systems [5,6].

DeepL Translator (DeepL GmbH, Cologne, Germany) is one of the MT applications employing neural network systems. It was launched in August 2017, and its Japanese translation service was launched in March 2020 [7]. DeepL Translator aims to provide accurate MT. However, the reliability of DeepL Translator in translating medical articles has not yet been verified. Therefore, in this study, we investigated the accuracy of MT from Japanese to English for a medical article using the DeepL Translator.

## Materials and methods

Subject

As the subject of this study, a medical article entitled “Dosimetric Comparison Between Carbon-Ion Radiotherapy and Photon Radiotherapy for Stage I Esophageal Cancer” was used [8]. This article was published in a MEDLINE-indexed, English peer-reviewed scientific journal. It describes a dose-volume analysis that is one of the major topics of interest in the field of radiation oncology. For this article, an original draft written in Japanese (hereafter “OJ”) is available.

Back translation and calculation of the match rate

**Figure 1:**
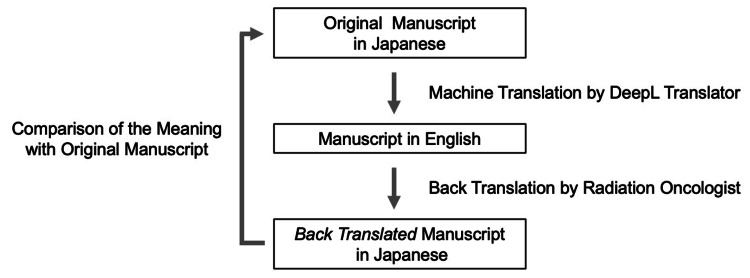
Overview of the study procedure.

Figure 1 shows an overview of the study design. The subject article (i.e., OJ) was translated from Japanese to English using the DeepL Translator in March 2021. The OJ was divided into four sections: Introduction, Materials and Methods, Results, and Discussion. MT using DeepL Translator was performed for each section. The automatically translated English text was back-translated into Japanese by translators [9,10]. The method of the back translation was as follows. An inappropriate word was translated as is. The translation was performed as literal translation, not free translation. If the translation was not possible, it was marked as “untranslatable.” The re-translated Japanese text was termed as the back-translated manuscript in Japanese (BTJ). In turn, the judges compared the OJ and BTJ for each sentence and determined whether the meanings were consistent. The number of sentences with matched meaning was counted, and the match rate of BTJ to OJ was calculated. The match rates were calculated for the entire text and each of the four sections, namely, Introduction, Materials and Methods, Results, and Discussion.

Translators and judges

Three translators individually performed the back translation from English to Japanese. The eligibility criteria for the translators were as follows: (i) a native Japanese speaker; (ii) a radiation oncology expert certified by the Japanese Society for Radiation Oncology; and (iii) five or more publications in English peer-reviewed scientific journals as the first author. Translators were not allowed to read the OJ in advance. Three judges individually performed their task. The judges met the following eligibility criteria: (i) a native Japanese speaker; (ii) not the translator of this study; and (iii) a radiation oncologist. The judges were blinded to the translators’ information.

Statistical analysis

Differences in match rates between each section were evaluated using the Mann-Whitney U test. The association between match rates and predictors was evaluated using logistic regression analysis. A p-value of <0.05 was considered statistically significant. Statistical analysis was performed using Stata software version 13.1 (StataCorp LLC, College Station, TX, USA).

## Results

Table 1 shows the match rates between OJ and BTJ. The total number of sentences examined was 121. The number of sentences in the Introduction, Materials and Methods, Results, and Discussion sections was 19, 31, 23, and 48, respectively. No sentences were determined by the judges to be untranslatable. The mean ± standard deviation (SD) of the match rate for all the sentences was 94.0 ± 2.9%. The mean ± SD of the match rate for the Results section was 100 ± 0%. The match rate in the Results section was significantly higher than the rates in the Introduction, Materials and Methods, and Discussion sections (p = 0.004, 0.001, and 0.001, respectively). The mean ± SD of match rate in the Materials and Methods section was 89.2 ± 3.9%. The match rate in the Materials and Methods section was significantly lower than the rates in the Introduction, Results, and Discussion sections (p = 0.028, 0.001, and 0.011, respectively). In the Introduction and Discussion sections, the match rates were not significantly different (p = 0.391).

**Table 1:** Match rate with the original manuscript in Japanese. SD: standard deviation

Section	Number of sentences	Match rate: mean ± SD (%)
Total	121	94.0 ± 2.9
Introduction	19	94.7 ± 5.3
Materials and Methods	31	89.2 ± 3.9
Results	23	100 ± 0
Discussion	48	94.2 ± 4.6

Among all the sentences in the BTJ, the total number of sentences determined to be discordant by two or more judges was 12. Among these, six had unclear subject and predicate statements in the OJ. There were three compound sentences in the OJ. The number of sentences with problems in English translation was three. The number of sentences with English translation errors was three, all of which were errors in the translation of technical terms. Back translation errors were found in two sentences.

Table 2 shows the results of the statistical analysis of the predictors for translation mismatch. Although the number of letters was not a significant predictable factor for a mismatch, a compound sentence was a significant predictor of mismatch in both univariate and multivariate analyses. Odds ratios [95% confidence interval (CI)] in univariate and multivariate analyses were 5.23 (1.52-18.02; p = 0.009) and 5.38 (1.06-27.31; p = 0.042), respectively. Sentences with unclear subject and predicate were significant predictors of mismatch in both univariate and multivariate analyses. Odds ratios (95% CI) in univariate and multivariate analyses were 11.11 (2.96-41.64; p < 0.001) and 7.60 (2.54-43.37; p = 0.001), respectively.

**Table 2:** Statistical analysis of predictors of translation mismatch. OR: odds ratios; CI: confidence interval

	Univariate			Multivariate		
	OR	(95% CI)	P-value	OR	(95% CI)	P-value
Number of letters	1.03	(0.99-1.07)	0.170	0.77	(0.15-4.08)	0.766
Compound sentences	5.23	(1.52-18.02)	0.009	5.38	(1.06-27.31)	0.042
Unclear subject and predicate	11.11	(2.96-41.64)	<0.001	7.60	(2.54-43.37)	0.001

## Discussion

This study investigated the accuracy of the English translation by DeepL Translator. The comparison between OJ and BTJ showed a high match rate of the meanings, suggesting high reliability of Japanese to English translation. To the best of our knowledge, this is the first study globally to investigate the accuracy of MT using DeepL Translator for medical articles.

The difference in the basic grammatical structure between two languages has been reported as a negative factor for the accuracy of conventional MT [2,3]. On the other hand, in this study, MT using DeepL Translator showed high accuracy even when the basic grammatical structure was different, such as that of Japanese and English. MT using DeepL Translator may ensure the accuracy of translation even when the basic grammatical structure of two languages differs.

Longer sentences have been reported to be a negative factor for the accuracy of MT [11]. In this study, there was no significant relationship between the length of the sentence and the accuracy of the translation; nevertheless, compound sentences and unclear subject and predicate were predictable factors for mismatch translations. Regarding the mistranslation of technical terms, it is imperative to use precise English words in OJ to avoid the mistranslation of technical terms. Therefore, to improve the accuracy of the translation by DeepL Translator, we recommend the following suggestions while writing OJ: clarify the subject and predicate, avoid using compound sentences as much as possible, and describe the English technical term, if necessary.

There are several limitations to this study, namely, the small sample size and subjective evaluation method. In addition, some translation errors cannot be detected by this research methodology. For example, some unsuitable words for scientific articles, such as “about” instead of “approximately,” are used in the translated manuscript; and words in passive and active voices are not consistent throughout the entire manuscript. To correct these errors, proofreading the translated English manuscript is crucial.

## Conclusions

In this study, DeepL Translator performed accurate MT for a medical article from Japanese to English. Compound sentences and unclear subject and predicate were significant negative predictors of translation accuracy in using DeepL Translator. DeepL Translator is expected to greatly reduce the barriers in academic writing in English for non-English-speaking researchers.
